# Ketoprofen Tolerance and Removal by *Rhodococcus* spp.

**DOI:** 10.3390/toxics14100845

**Published:** 2026-09-23

**Authors:** Maxim A. Polygalov, Vladimir V. Sorokin, Andrey L. Mulyukin, Irina B. Ivshina

**Affiliations:** 1 Institute of Ecology and Genetics of Microorganisms of the Ural Branch of the Russian Academy of Sciences, Perm Federal Research Center of the Ural Branch of the Russian Academy of Sciences, 13 Golev Str., 614081 Perm, Russia; 2 Winogradsky Institute of Microbiology, Research Center of Biotechnology, Russian Academy of Sciences, 60 let Oktyabrya, 7, bld. 2, 117312 Moscow, Russia; vlvlsorokin@gmail.com (V.V.S.); andlm@mail.ru (A.L.M.)

**Keywords:** nonsteroidal anti-inflammatory drugs, ketoprofen, pharmaceutical pollution, *Rhodococcus*, biotransformation, biodegradation, bacterial cell responses

## Abstract

Bacteria of the genus *Rhodococcus* are degraders of various ecotoxicants, including pharmaceuticals, yet their activity against ketoprofen—a non-steroidal anti-inflammatory drug (NSAID) frequently detected in aquatic environments—remains poorly explored. To address this gap, we assessed the tolerance and removal of ketoprofen across *Rhodococcus* strains from the Regional Specialised Collection of Alkanotrophic Microorganisms (official acronym IEGM, WFCC number 285). All 175 strains tolerated ketoprofen at minimum inhibitory concentrations far above reported environmental levels. Two particularly resilient strains, *R. erythropolis* IEGM 712 and *R. qingshengii* IEGM 746, sustained growth in ketoprofen-supplemented medium. Transmission electron microscopy (TEM) examinations showed that most cells preserved structural integrity and morphology, while energy dispersive X-ray spectroscopy and elemental mapping showed maintenance of K+ homeostasis. Over 14 days of cultivation in the mineral medium with n-hexadecane, these two strains removed 40–58% of ketoprofen. Exposure to ketoprofen altered cellular morphometry and surface charge (ζ-potential) and led to the formation of by-products with a predicted lower ecotoxicity than the parent compound. These findings underscore the potential of *Rhodococcus* strains for the bioremediation of ketoprofen.

## 1. Introduction

Pharmaceutical contamination of aquatic and terrestrial ecosystems is recognised as a major global environmental challenge [1,2,3]. Various drugs have been reported in polluted ecosystems at levels ranging from nano- to micrograms per litre, exerting proven or potential detrimental effects on flora and fauna [4]. As previously documented, the most prevalent pharmaceutical pollutants are antidepressants, hormonal drugs, antibiotics, and analgesics, including non-steroidal anti-inflammatory drugs (NSAIDs) [5,6,7,8,9,10]. These pollutants are continuously released into the environment and, when present together, can exert synergistic toxic effects [11,12]. It is clear that conventional wastewater treatment techniques are not effective for removing pharmaceutical active compounds [13,14,15,16]. Moreover, conventional degradation methods, such as filtration, flocculation, aeration, UV irradiation, and reverse osmosis, are costly, energy-consuming, and may form toxic by-products [15,16,17,18,19]. Consequently, environmentally safe and efficient bioremediation approaches are being considered as alternatives to physicochemical wastewater decontamination [3,20,21]. A promising avenue for the biodegradation of pollutants, including pharmaceuticals, is the utilisation of bacteria belonging to the genus *Rhodococcus* due to their powerful catabolic potential [22,23,24].

Bioremediation approaches for removing ketoprofen (2-(3-benzoylphenyl)propionic acid, CAS 22071-15-4), a widely used NSAID, are currently receiving significant attention. The presence of this drug in aquatic systems (groundwater, surface water, and wastewater) at concentrations ranging from nanograms to hundreds of micrograms (maximum) per litre has been reported, making it a key target for eco-pharmacovigilance [25,26,27]. Ketoprofen can induce oxidative stress in aquatic organisms, impair cellular signalling, reduce body size, decrease reproductive function and survival, and damage the liver, kidneys, and gills [28,29]. It can even result in the death of non-target organisms, including birds [30,31,32,33]. Moreover, the biotransformation of ketoprofen can generate products with photoallergenic properties [27].

Currently, the available data on the biodegradation of ketoprofen by microorganisms remains limited. The existing literature has predominantly focused on its degradation by fungi (e.g., *Trametes versicolor*, *Pleurotus ostreatus*) or pathogenic bacteria (*Raoultella* sp., *Pseudomonas* spp.) [34,35,36,37]. Due to intrinsic metabolic versatility and adaptabiltity [23,24], we hypothesised that bacteria of the *Rhodococcus* genus can cope with ketoprofen as they are already known for the ability to degrade various pollutants, including acetylsalicylic acid [38] and NSAIDs (diclofenac and ibuprofen [39,40]) and have displayed great potential in remediating pollutants (as will be mentioned in Section 4). To the best of our knowledge, it is unknown whether rhodococci tolerate ketoprofen and how they respond to ketoprofen exposure at different concentrations and nutritional conditions even under laboratory conditions. Furthermore, the removal of ketoprofen by *Rhodococcus* species has not been documented.

To address these gaps, this study aimed to select tolerant *Rhodococcus* strains and evaluate their capacity to remove ketoprofen. The experimental design included (i) minimal inhibitory concentration (MIC) assays to identify resilient strains; (ii) electron microscopy of cells grown with the drug; (iii) determination of optimal conditions of ketoprofen removal; (iv) analysis of the cell-free culture liquid for residual ketoprofen levels and byproducts; and (v) monitoring of morphological changes during exposure to ketoprofen.

## 2. Materials and Methods

### 2.1. Chemicals

Acetonitrile, orthophosphoric acid, and *n*-hexadecane with chemical and analytical purity were sourced from Cryochrome, Russia; Merck (Darmstadt, Germany), Germany; and Sigma-Aldrich (St. Louis, MO, USA), United States, respectively. Ultrapure water was produced using a Millipore Simplicity system (Merck, Darmstadt, Germany). Ketoprofen was used as a pure pharmaceutical substance (99.99% purity, odourless white powder, poorly soluble in water, BLD Pharmatech Ltd., Shanghai, China).

### 2.2. Microrganisms

In sum, 175 bacterial strains representing various *Rhodococcus* species (Table 1) were obtained from the Regional Specialised Collection of Alkanotrophic Microorganisms (official acronym IEGM, the World Federation for Culture Collections number 285, http://www.iegmcol.ru, accessed on 27 July 2026) [41].

Starter cultures were grown in LB broth with shaking at 160 rpm and 28 °C for 48 h. The optical density (OD_600_) was monitored on a Lambda EZ201 spectrophotometer (Perkin-Elmer, Shelton, CT, USA).

### 2.3. Assays of Minimum Inhibitory Concentrations

The minimum inhibitory concentrations (MICs) of ketoprofen were determined using the sequential dilution of bacterial cultures in LB broth with 96-well microplates (MiniMed, Moscow, Russia). A concentrated stock solution of ketoprofen was prepared in distilled water with 3 × 10^−2^ M NaOH and sterilised by passing through Labfil nylon 0.22 µm filters (China). The initial working ketoprofen concentration of 2.5 g L^−1^ was selected to asses any potential antimicrobial effect. Aliquots of the initial ketoprofen solution were added to the first row in each plate and serially diluted twofold to 0.3125 g L^−1^.

Bacterial suspensions for testing were prepared from 48 h cultures grown in LB broth. Cells were harvested by centrifugation (15 min, 3000× *g*), washed two times in 10 mM phosphate buffer (pH 7.0), and suspended in fresh LB broth to reach 5.0 × 10^8^ cells per mL. Each microplate well was inoculated with 10 µL of this suspension to 5.0 × 10^6^ cells mL^−1^. Microplates were incubated at 28 °C for 72 h on a Titramax shaker (Heidolph, Schwabach, Germany).

After incubation, 50 μL of 0.2% iodonitrotetrazolium chloride (INT, Sigma-Aldrich, United States) was added to wells, and microplates were incubated for a further 2 h to allow INT reduction by actively respiring cells to insoluble red-violet INT-formazan. Its formation was monitored spectrophotometrically at 630 nm using a Multiskan Ascent reader (Thermo Electron Corporation, Vantaa, Finland) and Ascent Software v. 2.6 (Thermo Labsystems, Vantaa, Finland).

### 2.4. Growth of Bacteria in the Presence of Ketoprofen

Bacteria were inoculated into LB broth containing ketoprofen at 10 µg L^−1^ or 10 mg L^−1^ and then incubated under aerated conditions at 28 °C for 120 h. The 10 µg L^−1^ concentration represents elevated environmental levels (review [25] and references therein). The 10 mg L^−1^ concentration corresponds to the approximate maximum plasma concentration (~10 µg mL^−1^) following therapeutic administration, according to the product information. Control cultures were incubated in ketoprofen-free LB under identical conditions.

### 2.5. Ketoprofen Removal

The strains were cultivated in light-shielded Erlenmeyer flasks (250 mL) with 100 mL of mineral medium RS of the following composition (g L^−1^): K_2_HPO_4_, 2.0; KH_2_PO_4_, 2.0; KNO_3_, 1.0; (NH_4_)_2_SO_4_, 2.0; NaCl, 1.0; MgSO_4_, 0.2; CaCl_2_, 0.02; FeCl_3_ × 7H_2_O, 0.001. Ketoprofen was added from the stock solution to amounts of 10 ng L^−1^ or 10 µg L^−1^, representing typical and elevated environmental levels, respectively [25]. Cultures were incubated at 28 °C with agitation at 160 rpm. Since ketoprofen does not support growth as the sole carbon and energy source, *n*-hexadecane 0.1% (*v*/*v*) was supplied as a co-substrate.

The controls were as follows: (1) sterile ketoprofen and *n*-hexadecane-supplemented RS medium to assess the abiotic degradation; (2) sterile RS medium with ketoprofen and heated *Rhodococcus* cells (autoclaved three times at 1 atm; 20 min) to evaluate adsorption of ketoprofen on bacterial cells; and (3) RS medium with *n*-hexadecane and live cells, but without ketoprofen to distinguish metabolites resulting from the co-substrate or cellular metabolism.

### 2.6. Determination of Ketoprofen and Its Degradation Products

Concentrations of ketoprofen in cell-free culture liquid samples were determined by high-performance liquid chromatography (HPLC) using an LC Prominence 20A chromatography system (Shimadzu, Kyoto, Japan) equipped with a Discovery^®^ C18 reversed-phase column (25 × 4.6 mm, 5 μm, Supelco, Bellefonte, PA, USA) and a diode-array detector (SPD-M20A). The mobile phase consisted of phosphate buffer (pH 3.5) and acetonitrile in a 60:40 (*v*/*v*) ratio. The eluent flow rate was 0.5 mL min^−1^, the column temperature was maintained at 30 °C, and detection was performed at 254 nm. The injection volume was 10 µL.

For the analysis of low-concentration ketoprofen samples, solid-phase extraction (SPE) was used prior to HPLC analysis. Briefly, 100 mL of sample was adjusted to pH 2.5–3.0 and loaded onto an Oasis HLB cartridge. Ketoprofen was eluted with methanol, evaporated under a gentle stream of nitrogen, and reconstituted in 1 mL of solvent, providing an approximately 100-fold preconcentration of the sample. The resulting extracts were analysed by HPLC at 254 nm.

A calibration range of 10–10,000 ng L^−1^ was used for the analysis. However, a formal analytical validation specifically covering the low-ng L^−1^ range was not performed in this study. Therefore, concentrations close to 10 ng L^−1^ should be regarded as approximate values rather than fully validated quantitative measurements.

Degradation products were identified by liquid chromatography–mass spectrometry (LC-MS) using a Shimadzu Prominence LC system (Shimadzu, Japan) equipped with a Phenomenex Luna 3u C18(2) 100A chromatographic column (150 × 3.0 mm) (Phenomenex Inc., Torrance, CA, USA). Elution was performed isocratically with a mobile phase consisting of 0.1% aqueous formic acid and acetonitrile (80:20, *v*/*v*) at a flow rate of 0.3 mL min^−1^. Mass-spectrometric detection was performed in positive ion scan mode within the *m*/*z* range of 50–500 using a DUIS interface. The flow rates of the nebulising, heating, and drying gases were set to 3.0 L min^−1^, 10.0 L min^−1^, and 10.0 L min^−1^, respectively. The temperatures of the interface, desolvation line, and heating block were maintained at 200 °C, 300 °C, and 400 °C, respectively. The capillary voltage was set to 4000 V with positive ionisation polarity.

### 2.7. Respiration Assay

The respiratory activity of bacterial cells was measured in a Micro-Oxymax^®^ respirometer (Columbus Instruments, Columbus, OH, USA). Samples were maintained at 28 °C ± 2 °C with continuous stirring (160 rpm) and oxygen concentrations were recorded over 14 days. Oxygen uptake rates were calculated from these measurements and expressed as µL h^−1^.

### 2.8. Microscopy

#### 2.8.1. Atomic Force Microscopy (AFM)

AFM-imaging was performed using an Asylum MFP-3D-BIO™ microscope (Asylum Research Inc., Santa Barbara, CA, USA) in tapping mode in air with an AC240TS silicon cantilever (Asylum Research Inc., Santa Barbara, CA, USA) (resonant frequency, 50–90 kHz; spring constant, 0.5–4.4 N m^−1^). Image processing was carried out in Igor Pro 6.22A (WaveMetrics, Portland, OR, USA).

#### 2.8.2. Transmission Electron Microscopy (TEM) Imaging

Specimens were prepared according to the protocol [40]. Briefly, washed cells were fixed with 2.5% (*w*/*v*) glutaraldehyde for 2.5 h and post-fixed in 1% (*w*/*v*) osmium tetroxide in sodium cacodylate buffer. The fixed material was sequentially dehydrated in a graded ethanol series up to absolute ethanol saturated with uranyl acetate. The material was embedded in epoxy resin and sectioned using an ultratome (LKB, Luleå, Sweden). Thin sections were stained with lead citrate and examined under a JEM-1400 microscope (JEOL, Tokyo, Japan).

#### 2.8.3. TEM with Energy Dispersive X-Ray Spectroscopy (EDX)

Unfixed cell suspensions in sterile water were dropped onto carbon-reinforced Formvar-coated copper grids. After air-drying, samples were analysed using a JEM-1400 TEM (JEOL, Japan) at an accelerating voltage of 80 kV and a 15° tilt angle. Elemental mapping and EDX spectra were acquired using an Inca Energy-350 EDXA system and AZtec software (both Oxford Instruments, Abingdon, UK).

### 2.9. Zeta-Potential Measurements

Cells were washed twice in KNO_3_ buffer and collected by centrifugation (HERMLE Z200A; Hermle, Gosheim, Germany). The pellets were resuspended in fresh buffer to an OD_600_ of 0.5 and monitored with a Lambda EZ201 spectrophotometer (Perkin-Elmer, Shelton, CT, USA). The electrokinetic (zeta, or ζ-) potential was recorded using a ZetaSizer Nano ZS analyser and Malvern ZetaSizer software v. 2.2 (Malvern Instruments, Malvern, UK). Calculation of the zeta-potential followed Equation (1).

(1) $$\zeta =\frac{\eta u}{\varepsilon_{0}\varepsilon_{r}}$$

where ζ is zeta-potential, V; u, electrophoretic mobility, m^2^/Vs; η, viscosity, N/m^2^s; ε_0_, dielectric permittivity in vacuum, F/m; and ε_r_, relative dielectric permittivity.

### 2.10. Re-Analysis of Genomic Data

The whole genome sequence of *R. qingshengii* IEGM 746 was deposited in DDBJ/ENA/GenBank (accession numbers JAJNDF010000001–JAJNDF010000078), with basic genomic features previously described [42].

### 2.11. In Silico Analysis of Ecotoxicity of Ketoprofen and Its Biodegradation Products

Ecotoxicity of ketoprofen and its derivatives was predicted using the ECOSAR (Ecological Structure Activity Relationships) within the EPI Suite software package (v. 4.11 EPA, Washington, DC, USA), and predicted endpoints were derived from ECOSAR’s structure–activity relationship and relevant toxicological database.

### 2.12. Statistical Analysis

The experiments on ketoprofen removal were performed on three independent biological replicates. Data were analysed using Excel 2021 (Microsoft, Redmond, WA, USA), and the mean and standard deviation were calculated. Statistical comparisons between control and ketoprofen-treated groups were performed using an unpaired Student’s *t*-test. A significance level of *p* < 0.05 was considered statistically significant, and *p* < 0.01 was considered highly significant. In the text, significance levels are indicated as *p* < 0.05 and *p* < 0.01, accordingly.

## 3. Results

### 3.1. Tolerance of Rhodococcus Strains to Ketoprofen

All 175 strains from the IEGM collection exhibited high tolerance to ketoprofen, as revealed by MIC assays. In LB broth, rhodococci under study withstood concentrations several orders of magnitude higher than the maximum levels reported in the environment [25], despite some strain-to-strain variations (Table 1). For 82% of the strains, the MIC exceeded 1,000,000 μg L^−1^, whereas *R. qingshengii* IEGM 746 and *R*. *erythropolis* IEGM 712 showed growth at the highest ketoprofen concentration tested (2,500,000 μg L^−1^), unlike the type strains of other *Rhodococcus* species. The strains IEGM 712 and IEGM 746 were selected for growth tests in LB broth supplemented with ketoprofen at 10 µg L^−1^ or at 10 mg L^−1^. Both strains demonstrated comparable growth in the drug-free and drug-supplemented media over a five-day period.

Thin-section TEM showed that most ketoprofen-exposed cells of *R. erythropolis* IEGM 712 (Figure 1) and *R. qingshengii* IEGM 746 (Figure 2) retained intact cell walls and intracytoplasmic structures, with only a small number showing signs of damage. In the presence of ketoprofen, some cells contained electron-transparent regions of varying dimensions within the cell wall. Cells in both the control and ketoprofen-supplemented cultures were surrounded by extracellular matter, appearing as outgrowths or net-like structures (Figure 1 and Figure 2).

TEM with EDX-spectroscopy and elemental mapping of *R. erythropolis* IEGM 712 and *R*. *qingshengii* IEGM 746 in washed and unfixed specimens showed that at both concentrations, ketoprofen exerted no effect on the intracellular pool and distribution of chemical elements in the majority of these bacteria. A negligible fraction of cells in cultures exposed to ketoprofen exhibited a decline in the K, P, and S levels, indicative of destructive processes (Figure 3).

Non-invasive TEM of washed, unfixed suspensions of the two rhodococci strains revealed numerous extracellular particles and blebs in close proximity to the cell wall and capsular layer (Figure 4). Furthermore, EDX spectroscopy of selected particles surrounding *R. erythropolis* IEGM 712 cells demonstrated the presence of biogenic elements, including phosphorus, sulphur, potassium, and magnesium, in contrast to the cell-free space (Figure 4). Thus, the electron microscopy examinations demonstrate that the majority of *R. erythropolis* IEGM 712 and *R. qingshengii* IEGM 746 cells remained intact when subjected to ketoprofen.

### 3.2. Removal of Ketoprofen from Aqueous Phase by the Selected Rhodococcus Strains IEGM 712 and IEGM 746

Culture supernatants were analysed periodically by HPLC to evaluate ketoprofen degradation. Over 14 days of cultivation in RS medium supplemented with *n*-hexadecane, *R. erythropolis* IEGM 712 and *R. qingshengii* IEGM 746 degraded 40–58% of ketoprofen. At an initial concentration of 10 µg L^−1^, strain IEGM 746 exhibited slightly higher removal of ketoprofen than strain IEGM 712. Conversely, strain IEGM 712 degraded ketoprofen with comparable efficiency at a lower concentration of 10 ng L^−1^ (Table 2). Biodegradation was slow and became evident only after 8 days of incubation. In the sterile control (ketoprofen in RS medium with *n*-hexadecane), the drug concentration remained constant throughout the experiment (Figure 5). It should be noted that the removal values obtained at the initial ketoprofen concentration of 10 ng L^−1^ should be considered as approximate, as a formal analytical validation specifically covering this low concentration range was not performed; these data are presented to illustrate the trend rather than to provide fully validated quantitative measurements. Nevertheless, in the heat-inactivated cell control, no significant decrease in ketoprofen concentration was observed over the 14-day incubation period, indicating that adsorption to bacterial cells did not contribute substantially to the removal of ketoprofen from the aqueous phase.

Data are presented as the mean of three biological replicates. Standard deviations were below 5% and have been omitted for clarity.

The values for the 10 ng L^−1^ samples were obtained after solid-phase extraction (SPE) and approximately 100-fold preconcentration. A formal analytical validation specifically covering the low-ng L^−1^ range was not performed. Therefore, these values should be regarded as approximate rather than fully validated quantitative measurements.

Supernatant samples from *R. qingshengii* IEGM 746 cultures grown for 14 days with 10 µg L^−1^ of ketoprofen were analysed by LC-MS. Ketoprofen degradation was accompanied by the formation of two intermediate products: 2-(3-(3-hydroxybenzoyl) phenyl)-propanoic acid and 2-[(3-hydroxy(phenyl)methyl)phenyl]-propanoic acid (Figure 6). The identified transformation products had the following mass-to-charge ratios (*m*/*z*) in positive ion mode: (2-(3-(3-hydroxybenzoyl)phenyl)propanoic acid), *m*/*z* 271 [M + H]^+^; (2-[(3-hydroxy(phenyl)methyl)phenyl]propanoic acid), *m*/*z* 257 [M + H]^+^. The proposed structures were consistent with those reported for ketoprofen degradation by *Trametes versicolor* [36] and *Bacillus paralicheniformis* [37]. ECOSAR predictions indicated that both metabolites were less ecotoxic than the parent drug. In particular, predicted toxicity towards aquatic invertebrates, fish, and green algae was reduced by 1.24- to 2.07-fold (Table 3).

### 3.3. Cell Activity and Morphological Changes in Rhodococci During Ketoprofen Degradation in Mineral Medium

The rate of oxygen consumption by *R. qingshengii* IEGM 746 in the RS medium supplemented with ketoprofen (10 µg L^−1^) reached a plateau after 18 h and remained high for up to 92 h. In ketoprofen-free medium, O_2_ uptake followed a similar pattern up to 43 h, after which it dropped sharply, stayed at a low plateau, and then rose to a moderate level. By 92 h, oxygen consumption by cells grown in the presence of ketoprofen was approximately 1.5-fold higher than in the control (Figure 7). Growth of strain 746 in the control and ketoprofen-containing media was accompanied by acidification and OD increments (Figure 8).

AFM analysis revealed proposed morphological changes in *R. qingshengii* IEGM 746 after 7 days of exposure to 10 µg L^−1^ ketoprofen (Figure 9). The most notable changes were a 2.3-fold reduction in cell length and a 1.3-fold reduction in width, accompanied by a 1.43-fold increase in the surface-area-to-volume ratio (Table 4). Ketoprofen exposure also promoted the appearance of extracellular fluid on day 7 (Figure 9b,d). Additionally, cells grown with the drug exhibited greater surface roughness and tendency to aggregate (Figure 9d).

Analysis of the ζ-potential showed a statistically significant increase in the negative surface charge of cells exposed to ketoprofen. The mean ζ-potential shifted by 19.322 mV, from −4.71 ± 0.13 mV in the control to −24.032 ± 0.164 mV in the ketoprofen group (Figure 10).

Thus, the exposure to ketoprofen in nutritionally poor medium was accompanied with alterations in cell morphology and surface charge.

## 4. Discussion

Bacteria of the genus *Rhodococcus* are renowned for their capacity to bioremediate organic and inorganic contaminants, offering a promising solution to significant ecological concerns [42,43,44]. Various *Rhodococcus* species efficiently and almost completely degrade diverse pyrethroid insecticides under laboratory conditions and remediate contaminated soils [45,46,47]. Specifically, *R. qingshengii* strain djl-6—a representative of the same species analysed in our study—demonstrates strong potential for cleaning up soils polluted with the widely used fungicide carbendazim [48]. Immobilised bacteria, including strain djl-6, are also practically relevant for the effective removal of multiple pesticides from soil and water [49].

Prior to our study, the removal of the pharmaceutical active compound ketoprofen by *Rhodococcus* species had not been demonstrated, although their ability to degrade other pharmaceuticals, such as diclofenac and ibuprofen, was already known [39,40]. It is a novel finding that many *Rhodococcus* strains tolerate ketoprofen at exceptionally high concentrations (Table 1), several orders of magnitude above environmental levels [25]. Although ketoprofen causes known or predicted harm to organisms in natural ecosystems [28,29,30,31,32,33], information on its effects on microorganisms remains limited. This tolerance is consistent with the resilience of *Rhodococcus* and taxa that are phylogenetically related to other NSAIDs [39,50]. Notably, *R. qingshengii* IEGM 746, isolated from oil-polluted soil, utilises *n*-hexadecane and crude oil effectively. Isolates from such biotopes commonly possess potential for xenobiotic degradation [3,23,24].

The removal of ketoprofen by *R. qingshengii* IEGM 746 (Table 2) is comparable to that of *Bacillus paralicheniformis* (59%) [37]. However, *R. qingshengii* and *R. erythropolis* may offer biosafety advantages. The strains are non-pathogenic saprotrophs, whereas some *B. paralicheniformis* strains can act as opportunistic pathogens under specific conditions [51,52]. In our study, successful optimisation occurred at ketoprofen concentrations of 10 ng L^−1^ and 10 µg L^−1^ (Table 2) in mineral medium with *n*-hexadecane, whereas our preliminary experiments showed a removal efficiency of no more than 30% at 100 µg L^−1^. In comparison, the fungus *Trametes versicolor* reduced >80% ketoprofen after 21 days in a synthetic medium supplemented with yeast extract, while other fungi, including *T. reesei*, proved inefficient [34]. The rate of near-complete ketoprofen degradation by *T. versicolor* in a glucose-supplemented synthetic medium was also dependent on the initial drug concentration [36]. The products of ketoprofen generated by *R. qingshengii* IEGM 746—2-(3-(3-hydroxybenzoyl)phenyl)propanoic and 2-[(3-hydroxy(phenyl)methyl)phenyl] propanoic acids (Figure 6)—were predicted to be 1.2–2.0-fold less ecotoxic than parent compound (Table 3). The hydroxylated metabolites correspond to ketoprofen degradation products for *T. versicolor* [36] and *B. paralicheniformis* [37], suggesting conservation of the initial degradation steps.

It is important to acknowledge the analytical limitations of this study. Although SPE with approximately 100-fold preconcentration was used for the analysis of low-concentration ketoprofen samples, a formal analytical validation specifically covering the low-ng L^−1^ range was not performed. Therefore, the removal values obtained at an initial concentration of 10 ng L^−1^ should be regarded as approximate rather than fully validated quantitative measurements. These data illustrate the trend of ketoprofen removal at environmentally relevant concentrations, but further method validation, including determination of LOD/LOQ and spike-recovery experiments in the low-ng L^−1^ range, is required to confirm these observations.

It should be noted that the possible intracellular accumulation of ketoprofen was not assessed, and a complete mass balance was not established. Therefore, the observed decrease in the supernatant concentration is attributed primarily to biotransformation, although the possibility of minor intracellular uptake cannot be entirely excluded.

It should also be emphasised that this study demonstrates biotransformation rather than complete mineralisation of ketoprofen. Since ketoprofen does not support growth as the sole carbon and energy source, *n*-hexadecane was supplied as a co-substrate, and the observed removal is therefore co-metabolic. No CO_2_ evolution or dissolved organic carbon measurements were performed, and complete mineralisation was not assessed. Furthermore, the reduced ecotoxicity of the identified transformation products is based on ECOSAR predictions and has not been experimentally confirmed by bioassays (e.g., using *Daphnia*, algae, or bacterial toxicity tests).

Electron microscopy (Figure 1 and Figure 2) demonstrated that the most cells of the strains IEGM 712 and IEGM 746 remained intact during growth in rich LB medium in the presence of ketoprofen even at concentrations far exceeding the environmental levels. These observations align with previous reports on microbial response to other NSAIDs, such as meloxicam and ibuprofen [40,51]. It is a novel finding that the strains IEGM 712 and IEGM 746 showed a notable abundance of extracellular particles and vesicles under both control conditions and ketoprofen-exposed conditions. These structures were clearly visible in unfixed preparations using TEM (Figure 4). However, the multistage procedures typically used for thin-sectioning TEM (pelleting, chemical fixation and dehydration) are not well suited to visualising such extracellular particles.

The substantial potassium enrichment of extracellular structures relative to the surrounding milieu (Figure 4) indirectly suggests the presence of a membrane, consistent with extracellular vesicles (EVs), and the maintenance of a K^+^ gradient. Although EVs have been described in both Gram-negative and Gram-positive bacteria [53], evidence for EV production in *Rhodococcus* is limited. Surface-associated vesicles have been described in *Rhodococcus equi* [54] and were observed in our AFM images (Figure 9). Further work is needed to determine the nature, structure, composition, and functional roles of EVs in biotechnologically relevant *Rhodococcus* strains, such as IEGM 746. In particular, characterising EV membrane components, including ion transporters, is important given reports of non-bacterial EVs that contain a large-conductance calcium-activated potassium channel critical for ion homeostasis and vesicle stability [55].

Although MIC assays in rich LB broth (Table 2) showed that many *Rhodococcus* strains tolerate high ketoprofen concentrations, these results do not predict bioremediation potential in an oligotrophic mineral medium. Strains IEGM 712 and IEGM 746 (which grew in LB broth at the highest ketoprofen concentrations) were chosen first for removal experiments in the minimal medium with *n*-hexadecane, though other strains may also be useful. For strain IEGM 746, 10 µg L^−1^ of ketoprofen did not affect early growth dynamics or respiratory activity (Figure 7), despite changes in cell size and surface area (Figure 9 and Figure 10; Table 4). However, preliminary Live/Dead staining indicated that 100 µg L^−1^ of ketoprofen slightly increased the proportion of cells with damaged membranes compared to the control mineral medium.

Morphometric and surface-topology changes in IEGM 746 during exposure to ketoprofen in the minimal medium largely resemble those reported for other *Rhodococcus* species [56] and related bacteria [50]. Notably, ketoprofen-exposed *R. qingshengii* IEGM 746 produced extracellular fluid (Figure 9). These may contain biosurfactants that facilitate ketoprofen utilisation, as reported in sludge hydrolysis systems [57].

Re-analysis of the genomic data for strain IEGM 746 [42] revealed six genes (Table 5) encoding alkane-1-monooxygenases that act on the added co-substrate, *n*-hexadecane, whereas *R*. *erythropolis* and *R. pyridinivorans* possess four and two copies of this gene, respectively [58].

The genome of the strain IEGM 746 contains coding sequences (CDS) for monooxygenases, dioxygenases, proteins with decarboxylase activity, peroxidases, multicopper oxidases, vanillate O-demethylase oxidoreductases, and dehydrogenases [42]. However, identification of the specific enzymes implicated in ketoprofen transformation requires further corroboration to provide functional evidence and establish a link between enzyme activity and these genes. Studies on xenobiotic degradation by *Rhodococcus* species have successfully identified and directly demonstrated the functional roles of several enzymes involved in efficient biodegradation. These include amidase (PzcH), implicated in phenazine-1-carboxamide transformation [59]; *p*-nitrophenyl esterase (PnbA1564), which hydrolyzes prallethrin [45]; hydrolase (IpcH), active toward various carbamate insecticides [60]; hydrolase (CymE1), which converts cypermethrin [47]; and carboxylesterase (DcyR), active toward D-cyphenothrin [46]. Notably, the *cbe*H gene, encoding for hydrolase CbeH that catalyses the cleavage of C-O, C-C, and C-N bonds in some herbicides and aromatic pollutants, is widely distributed across α- and γ-Proteobacteria and *Rhodococcus* genera inhabiting polluted environments [61].

## 5. Conclusions

Numerous *Rhodococcus* strains from the IEGM collection (WFCC number 285) displayed high tolerance to ketoprofen at concentrations far exceeding environmental levels. Two particularly resilient strains, *R. erythropolis* IEGM 712 and *R. qingshengii* IEGM 746, removed from 40% to 58% of ketoprofen in a nutrient-poor mineral medium over 14 days. Notably, *R. qingshengii* IEGM 746 transformed ketoprofen into derivatives with lower predicted ecotoxicity. These findings highlight *Rhodococcus* species as promising candidates for the bioremediation of ketoprofen-contaminated wastewater; however, complete mineralisation was not confirmed, and the reduced ecotoxicity is based on in silico predictions that require experimental validation. Future studies should focus on elucidating the enzymatic mechanisms of ketoprofen transformation, including functional validation of candidate genes, and on assessing the mineralisation of the drug. Further research is also needed to evaluate the performance of these strains under real wastewater conditions, including the presence of competing substrates and microbial communities, and to confirm the reduced ecotoxicity of transformation products through experimental bioassays. Addressing these challenges will be essential for the practical application of *Rhodococcus* strains in ketoprofen bioremediation.

## Figures and Tables

**Figure 1:**
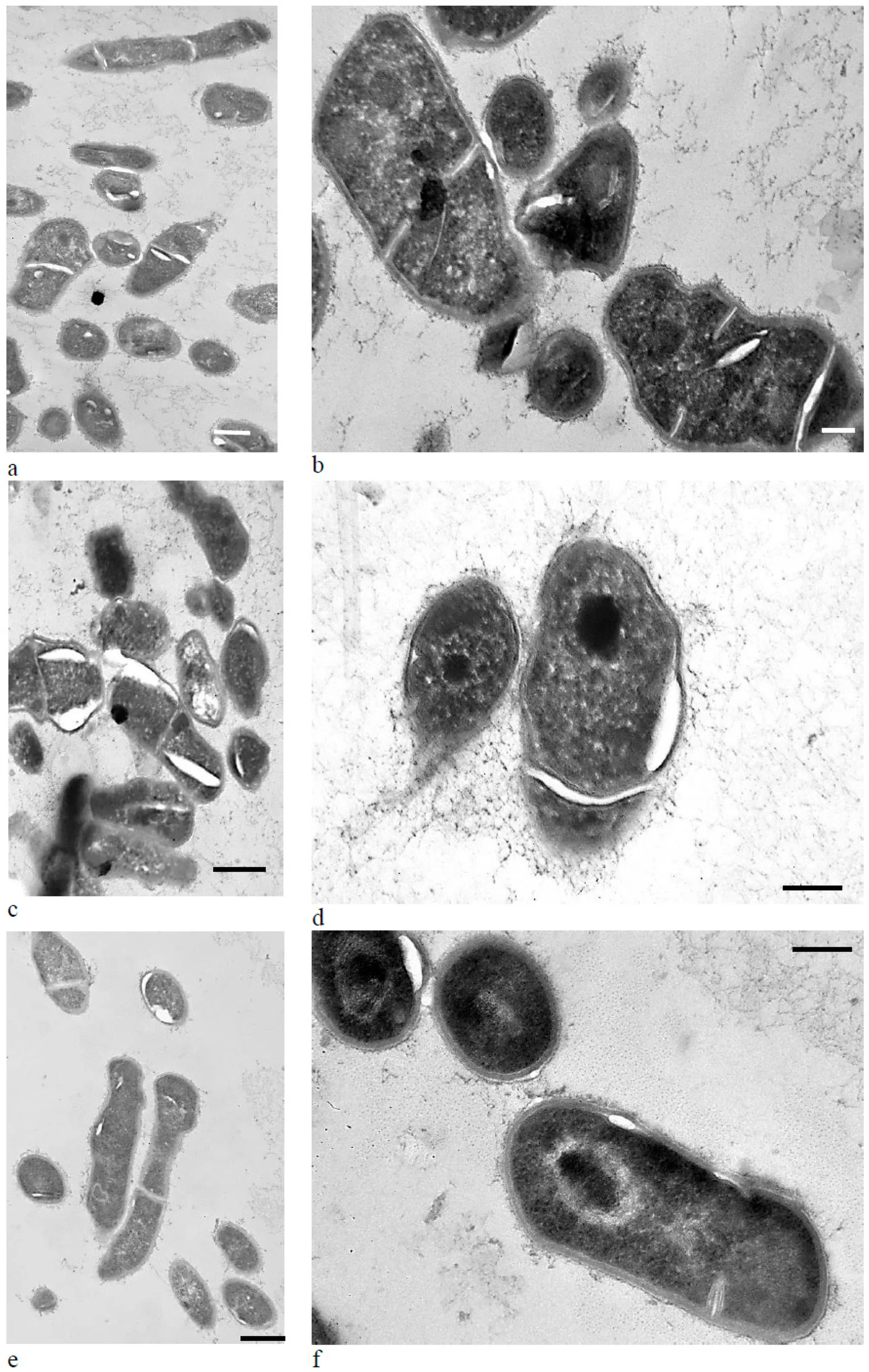
Transmission electron microscopy (TEM) images of *R. erythropolis* IEGM 712 cells grown in LB broth: (**a**,**b**) without the drug; (**c**–**f**) with ketoprofen in concentrations of (**c**,**d**) 10 µg L^−1^ or (**e**,**f**) 10 mg L^−1^. Bars: (**a**,**c**,**e**) 500 nm; (**b**,**d**,**f**) 200 nm.

**Figure 2:**
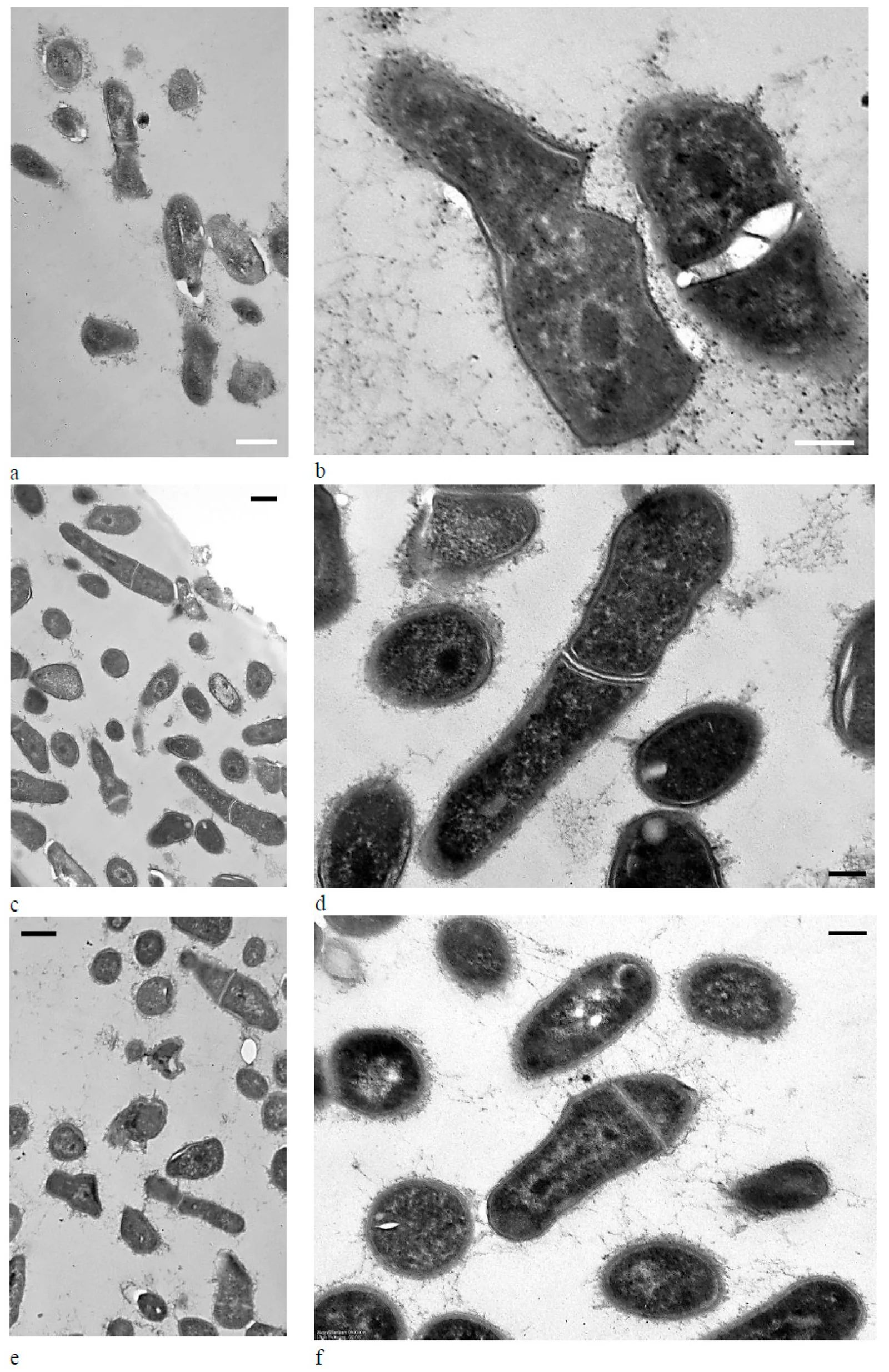
TEM images of *R. qingshengii* IEGM 746 cells grown in LB broth: (**a**,**b**) without the drug; (**c**–**f**) with ketoprofen in concentrations of (**c**,**d**) 10 µg L^−1^ or (**e**,**f**) 10 mg L^−1^. Bars: (**a**,**c**,**e**) 500 nm; (**b**,**d**,**f**) 200 nm.

**Figure 3:**
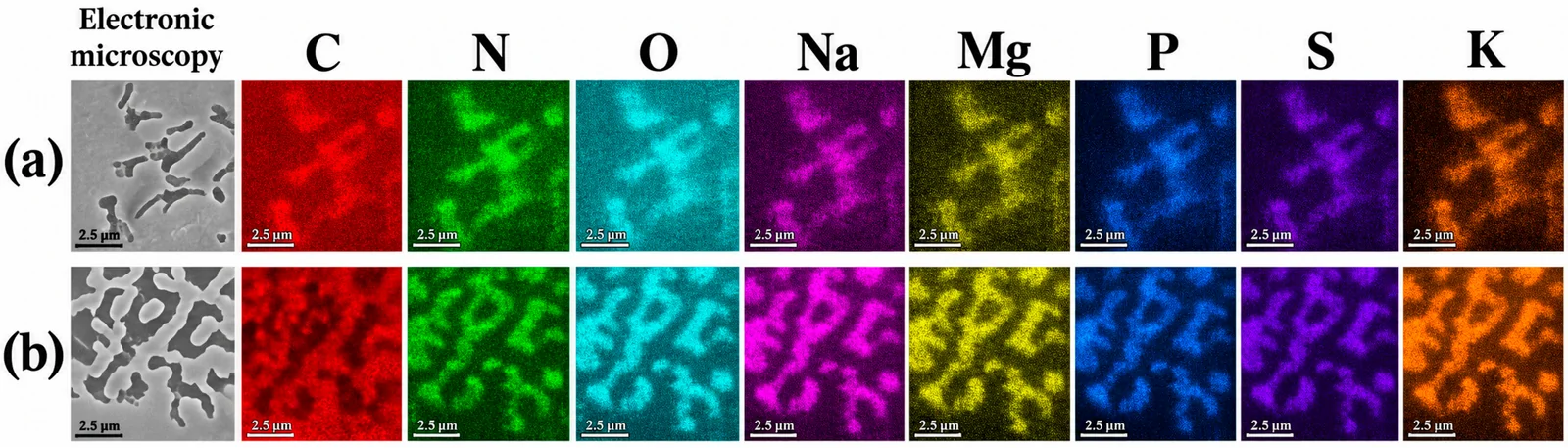
The elemental composition of *R. qingshengii* IEGM 746 cells, determined by mapping using X-ray microanalysis: (**a**) without ketoprofen; (**b**) in the presence of 10 micrograms/L of ketoprofen.

**Figure 4:**
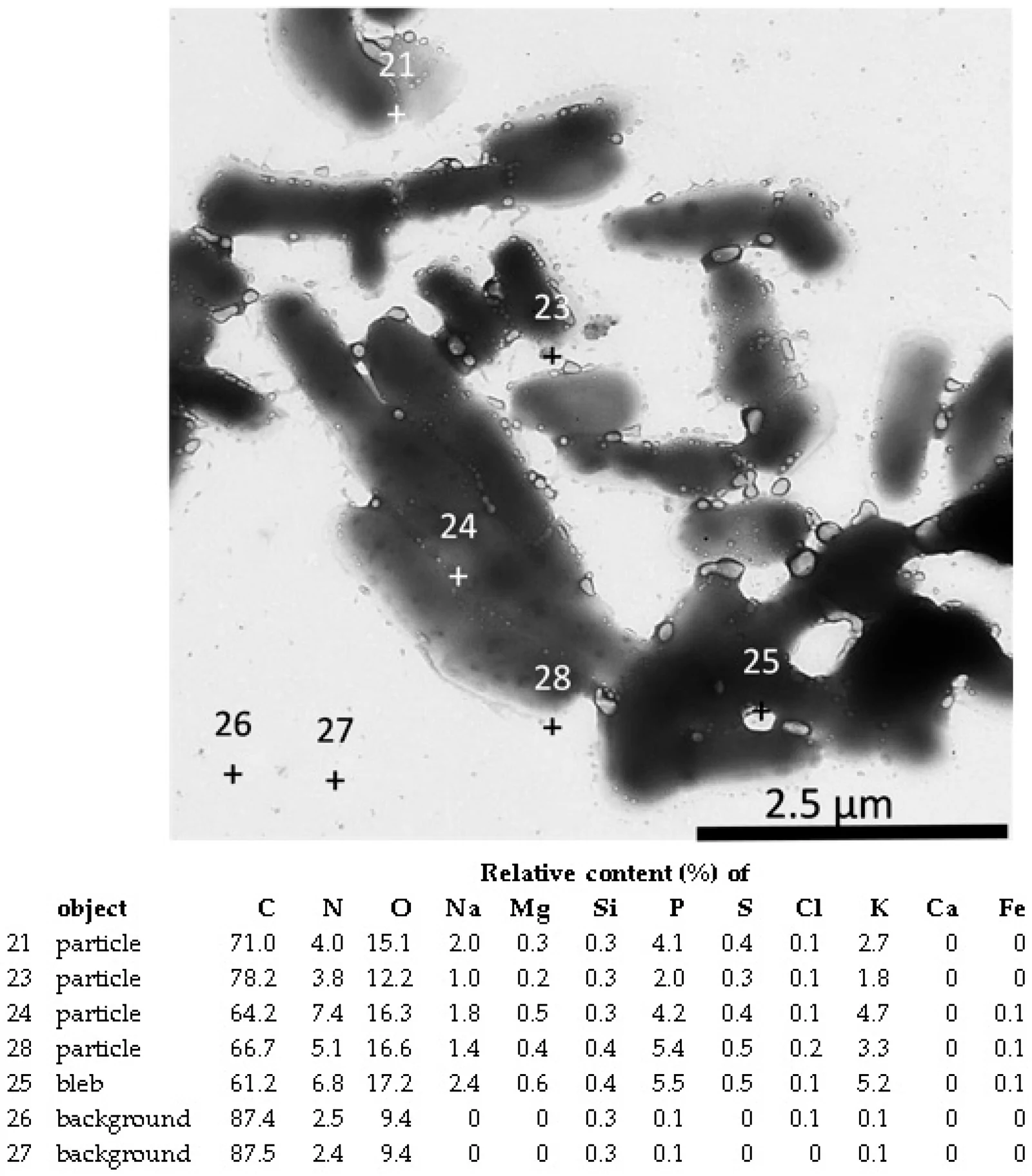
TEM image of *R. erythropolis* IEGM 712 (grown in LB broth with ketoprofen at 10 µg L^−1^) and the relative content (determined by means of EDX spectroscopy) of chemical elements in selected particles and zones around cells. Sites of X-ray probing are marked with +. Bar, 2.5 µm. Carbon and oxygen signals in the background areas 26 and 27 (milieu) arise from Formvar coating on grids with specimens.

**Figure 5:**
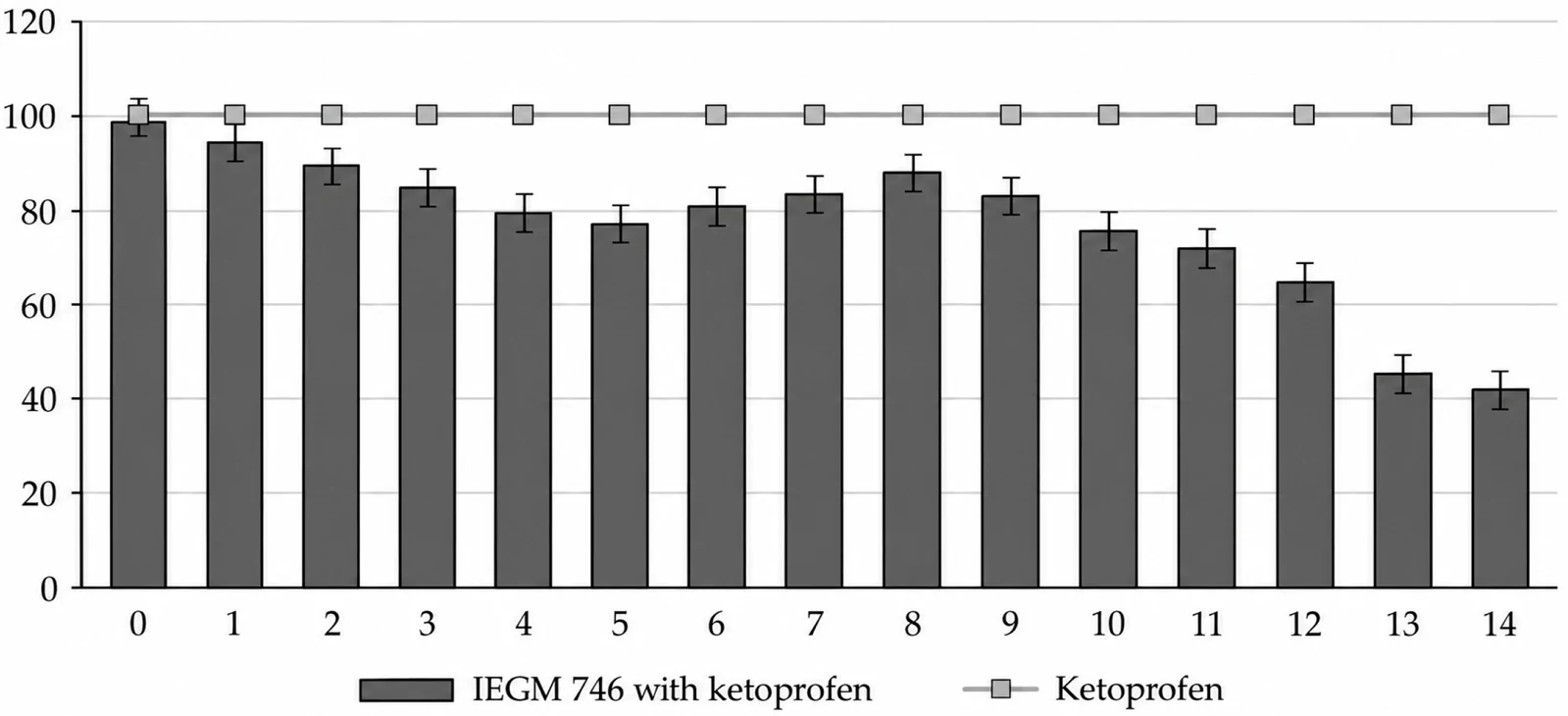
Removal of ketoprofen at an initial concentration of 10 ng L^−1^ in the culture medium of strain IEGM 746.

**Figure 6:**
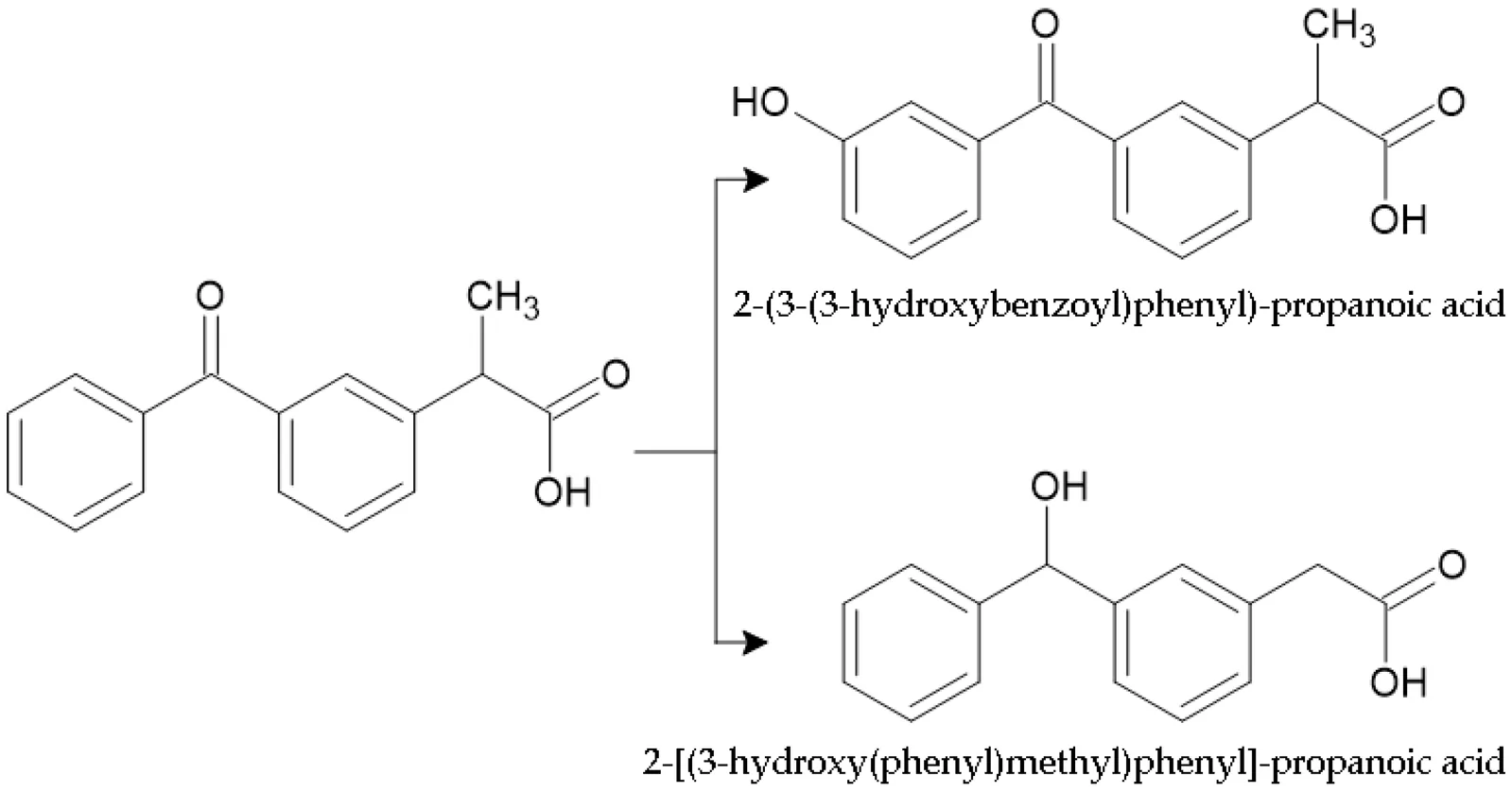
Structure of ketoprofen degradation products.

**Figure 7:**
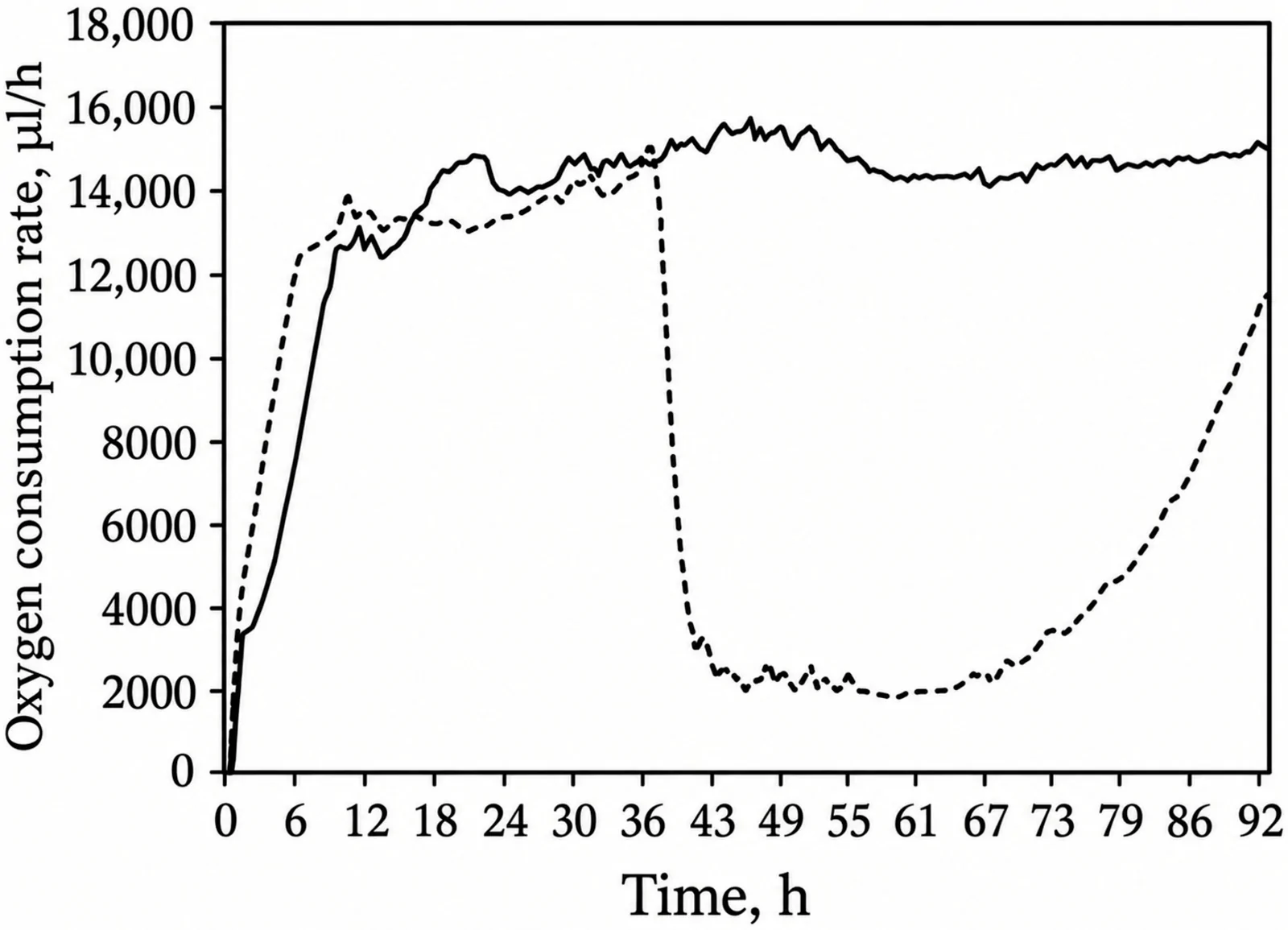
The rate of O_2_ consumption by *R. qingshengii* IEGM 746 in the presence of ketoprofen at 10 µg L^−1^ (---) and without the drug (–––) during the first 92 h of cultivation.

**Figure 8:**
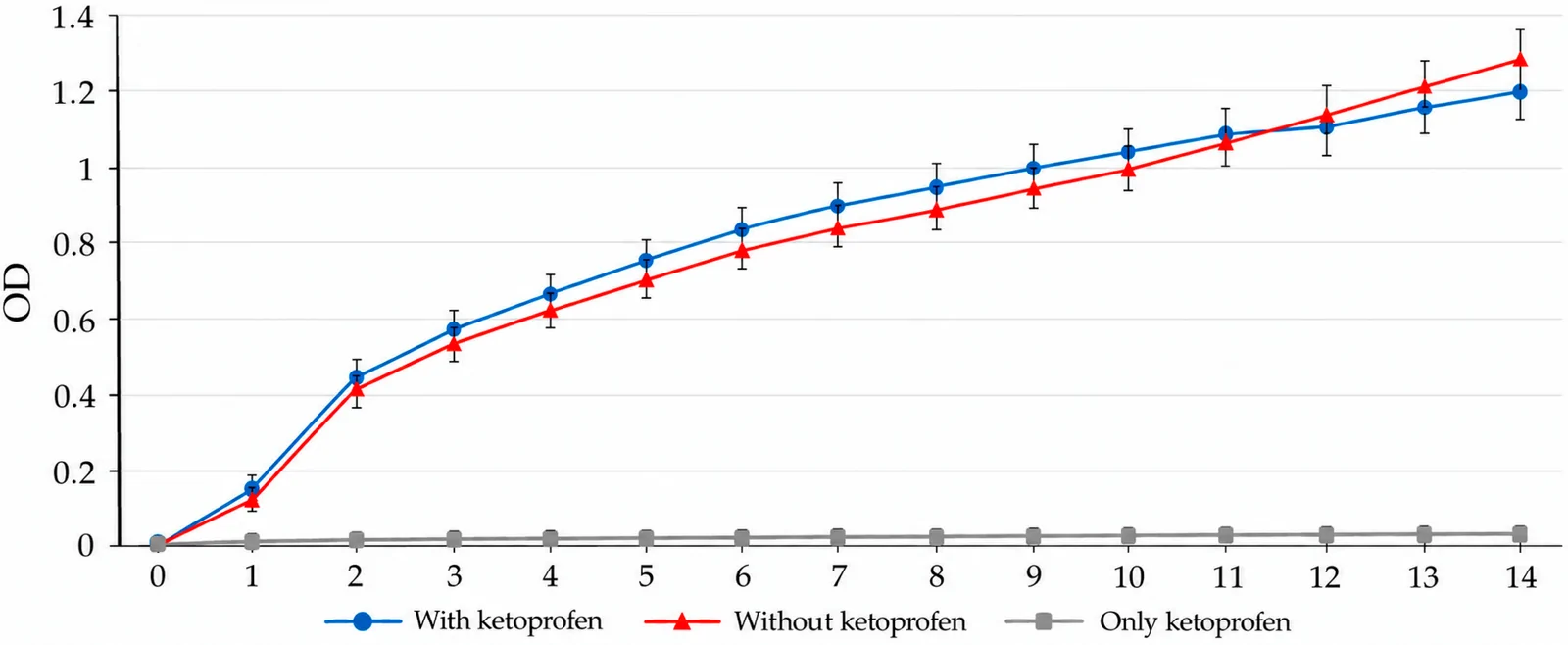
Curves of OD changes.

**Figure 9:**
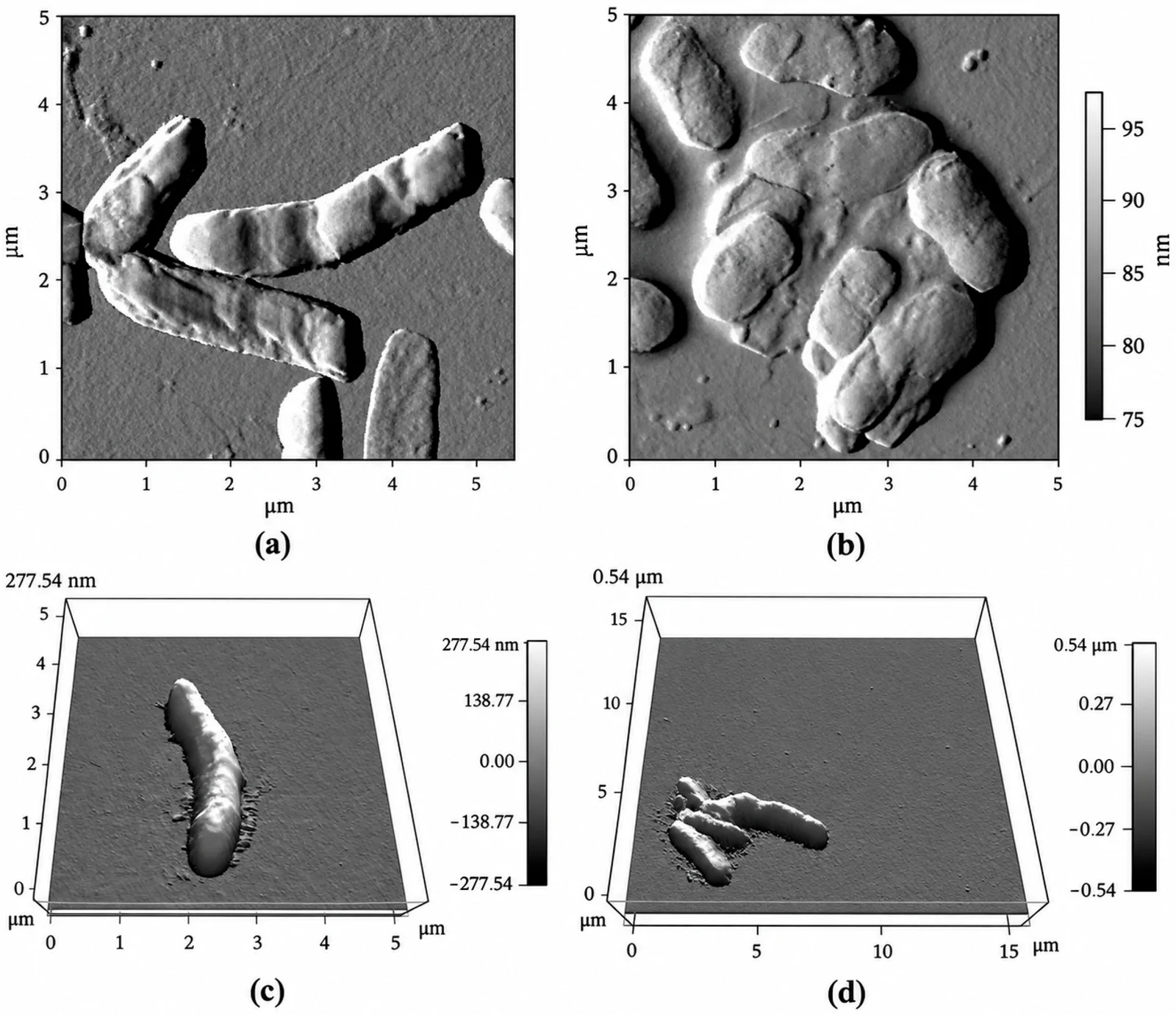
Atomic force microscopy (AFM) topography of *R. qingshengii* IEGM 746 cells after 7 days of cultivation in (**a**,**c**) control RS medium and (**b**,**d**) RS medium with ketoprofen (10 µg L^−1^). Panels display the surface topography (upper row) and 3D morphology (lower row). Arrows indicate blebs on the cellular surface.

**Figure 10:**
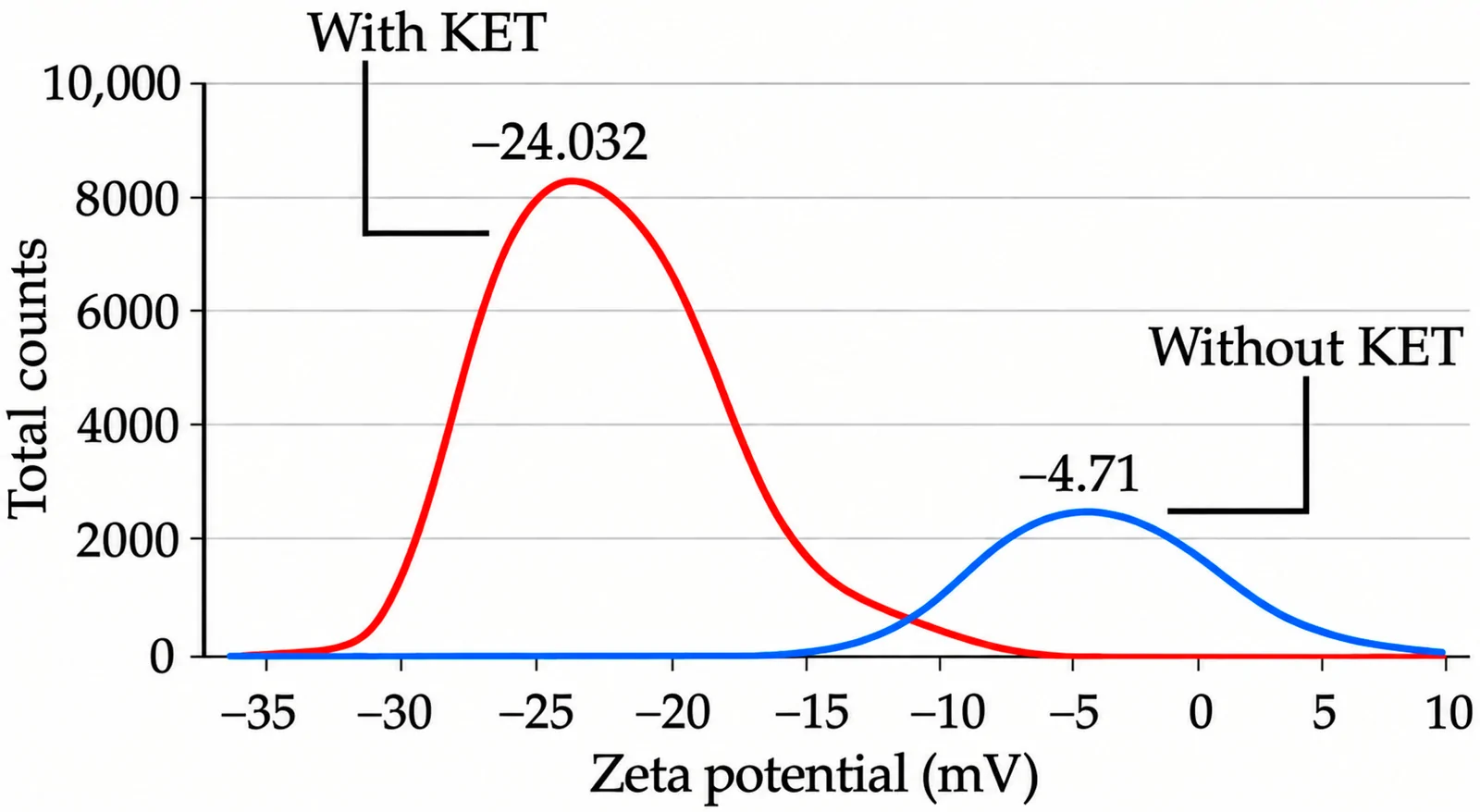
Zeta-potential shifts in *R. qingshengii* IEGM 746 cells cultured in RS medium in the presence and absence of 10 μg L^−1^ ketoprofen.

**Table 1:** *Rhodococcus* strains and minimum inhibitory concentrations (MICs) of ketoprofen.

Species	Strain	MIC, g L^−1^
*R. qingshengii*	IEGM 746 *	>2.5
*R. erythropolis*	IEGM 712	
*R. aetherivorans*	IEGM 911^T^, IEGM 1250	2.5
*R. erythropolis*	IEGM 24, IEGM 180, IEGM 185, IEGM 190, IEGM 193, IEGM 198, IEGM 214, IEGM 505, IEGM 506, IEGM 587, IEGM 588, IEGM 696, IEGM 703, IEGM 1179, IEGM 1189, IEGM 1321, IEGM1384, IEGM 1389, IEGM 1393	
*R. imtechensis*	IEGM 940^T^	
*R. pyridinivorans*	IEGM 1055^T^, IEGM 1142, IEGM 1227	
*R. qingshengii*	IEGM 247, IEGM 1016^T^	
*R. rhodochrous*	IEGM 66, IEGM 107, IEGM 608, IEGM 609, IEGM 629, IEGM 632, IEGM 646, IEGM 1360	
*R. ruber*	IEGM 70^T^, IEGM 71, IEGM 76, IEGM 85, IEGM 86, IEGM 90, IEGM 219, IEGM 220, IEGM 221, IEGM 222, IEGM 231, IEGM 235, IEGM 237, IEGM 320, IEGM 321, IEGM 322, IEGM 325, IEGM 326, IEGM 333, IEGM 341, IEGM 342, IEGM 343, IEGM 345, IEGM 346, IEGM 347, IEGM 348, IEGM 351, IEGM 352, IEGM 369, IEGM 371, IEGM 374, IEGM 375, IEGM 379, IEGM 380, IEGM 381, IEGM 382, IEGM 383, IEGM 384, IEGM 385, IEGM 386, IEGM 436, IEGM 437, IEGM 440, IEGM 442, IEGM 451, IEGM 453, IEGM 457, IEGM 483, IEGM 573, IEGM 596, IEGM 601, IEGM 615, IEGM 628, IEGM 1133	
*R. aetherivorans*	IEGM 1367	1.25
*R. cerastii*	IEGM 1243, IEGM 1278	
*R. cercidiphylli*	IEGM 1184	
*R. corynebacterioides*	IEGM 1202	
*R. erythropolis*	IEGM 7^T^, IEGM 10, IEGM 22, IEGM 23, IEGM 179, IEGM 192, IEGM 199, IEGM 212, IEGM 245, IEGM 251, IEGM 499, IEGM 504, IEGM 690, IEGM 711, IEGM 1020	
*R. fascians*	IEGM 34, IEGM 278	
*R. globerulus*	IEGM 685, IEGM 1019, IEGM 1203	
*R. koreensis*	IEGM 962^T^	
*R. maanshanensis*	IEGM 1396	
*R. qingshengii*	IEGM 267, IEGM 699, IEGM 1262, IEGM 1270, IEGM 1359	
*R. rhodochrous*	IEGM 654	
*R. ruber*	IEGM 65, IEGM 73, IEGM 77, IEGM 79, IEGM 80, IEGM 82, IEGM 83, IEGM 84, IEGM 91, IEGM 92, IEGM 172, IEGM 338, IEGM 387, IEGM 438, IEGM 439, IEGM 446, IEGM 452, IEGM 474, IEGM 485, IEGM 633	
*R. cercidiphylli*	IEGM 1322	0.625
*R. corynebacterioides*	IEGM 929^T^	
*R. erythropolis*	IEGM 183, IEGM 186, IEGM 201, IEGM 213, IEGM 215, IEGM 244, IEGM 252, IEGM 681, IEGM 701, IEGM 766, IEGM 1303	
*R. jostii*	IEGM 60, IEGM 508	
*R. ruber*	IEGM 328	
*R. wratislaviensis*	IEGM 1112^T^, IEGM 1171	
*R. yunnanensis*	IEGM 1323	
*R. cerastii*	IEGM 1327	0.3125
*R. corynebacterioides*	IEGM 931	
*R. erythropolis*	IEGM 197, IEGM 202, IEGM 250	
*R. globerulus*	IEGM 591^T^	
*R. percolatus*	IEGM 1031^T^	
*R. ruber*	IEGM 657, IEGM 759, IEGM 896	
*R. zopfii*	IEGM 673^T^	

* We consider this strain to belong to *Rhodococcus qingshengii*. Although *R. qingshengii* is a synonym for *R. erythropolis* (https://lpsn.dsmz.de/species/rhodococcus-qingshengii, last accessed on 22 July 2026) and IEGM 746 has previously been referred to as *R. erythropolis* IEGM 746 ([42]), it differs from *R. erythropolis* IEGM 746 strains deposited in the IEGM Collection. In particular, IEGM 746 shows the highest ANI value of 98.64% (https://www.ncbi.nlm.nih.gov/datasets/genome/GCF_021026075.1/, last accessed on 22 July 2026) and dDDH of 89.50% relative to *R. qingshengii* JCM 15477 and does not utilise inulin, maltose, mannitol, sorbitol, and trehalose, which are characteristic of *R. qingshengii* but not of *R. erythropolis* strains capable of using these substrates. The name *Rhodococcus qingshengii* is valid and can be used in place of *R. erythropolis* (https://lpsn.dsmz.de/species/rhodococcus-qingshengii, last accessed on 27 July 2026).

**Table 2:** Removal of ketoprofen in the RS medium inoculated with *Rhodococcus* strains.

Strain	Residual Ketoprofen Concentration, % from			
	Starting 10 ng L^−1^		Starting 10 µg L^−1^	
	by Day 7	by Day 14	by Day 7	by Day 14
*R. erythropolis* IEGM 712	91	51	94	60
*R. qingshengii* IEGM 746	84	42	89	54

**Table 3:** Predicted ecotoxicity of ketoprofen and products of its bacterial conversion.

Compound	Non-Mammals		Fish		Green Algae	
	ED_50_, 48 h	LD_50_, 48 h	ED_50_, 48 h	LD_50_, 48 h	ED_50_, 48 h	LD_50_, 48 h
Ketoprofen	16.58	937.52	23.4	997.61	10.31	17.87
2-(3-(3-hydroxybenzoyl)phenyl)-propanoic acid	34.28	1278.51	30.66	1774.2	14.54	36.32
2-[(3-hydroxy(phenyl)methyl)phenyl]-propanoic acid	31.94	1167.76	29.45	1710.17	14.1	30.02

**Table 4:** Morphometry changes due to the effect of ketoprofen (7 days).

Growth	Length, µm	Width, µm	Surface Area, S, µm^2^	Volume, V, µm^3^	S/V, µm^−1^
In the control RS medium	2.80 ± 0.350	1.15 ± 0.250	16.71 ± 0.670	4.19 ± 0.035	3.99 ± 0.170
In the presence of ketoprofen 10 µg L^−1^	1.20 ± 0.150	0.90 ± 0.200	6.78 ± 0.200	1.19 ± 0.006	5.70 ± 0.200

**Table 5:** Alkane-1-monooxygenase encoding genes in the *R. qingshengii* IEGM 746 genome.

Gene No.	Contig ID	Gene Location	Amplicon Size, bp
1	JAJNDF010000003.1	433,184–432,009	1175
2	JAJNDF010000004.1	551,343–552,509	1166
3	JAJNDF010000005.1	300,904–302,130	1226
4	JAJNDF010000005.1	509,037–510,197	1160
5	JAJNDF010000010.1	1466–315	1151
6	JAJNDF010000014.1	72,770–73,915	1145

## Data Availability

The raw data supporting the conclusions of this article will be made available by the authors on request.
